# Buying high quality, affordable, near-vision spectacles

**Published:** 2026-03-12

**Authors:** Samit Sakib Gore

**Affiliations:** 1 Director of Operations & Innovation: Vision Friend Sakib Gore, Mumbai, India.

**Near-vision spectacles need to be affordable, without sacrificing optical quality or durability.**

There are a range of different spectacle types available for the correction of presbyopia, including prescription near-vision spectacles, ready-made near-vision spectacles, bifocals, and progressive addition lenses. Of these, ready-made near-vision spectacles, which have the same dioptre lens for both eyes, are by far the most affordable and readily available option.

The **WHO Summary Guide on Quality Standards for Spectacles**^1^ makes recommendations regarding ophthalmic lenses and frames, ready-made spectacles for distance and near vision, and relevant ISO standards. Included in this is a recommendation that near-vision ready-made spectacles are marked with the spherical power, which allows users and clinicians to easily determine the strength of the lenses without the use of a focimeter. Depending on your region, additional quality standards may apply.

In contexts where prescription spectacles are provided, the quality requirements are naturally more complex, from the skill of the optometrist performing refraction, to the production of the prescription lenses. These components can be assessed using the **Q.REC (Quality of Refractive Error Care)** toolkit.^2^

Ready-made near-vision spectacles can be purchased from bulk suppliers and shipped via air or sea freight. In an informal review of six bulk optical suppliers in China in 2024, prices ranged from USD 0.43 to over USD 1.00 per pair, depending on supplier and purchasing volume. In India, 2024 prices from one of the largest wholesalers in-country ranged from USD 0.35-0.80 per pair.

## Buyer's guide to presbyopia spectacles

The frames should be of good enough quality to be comfortable and to reduce the likelihood of accidental breakage.

Due to the natural progression of presbyopia, near-vision spectacles will need to be replaced at regular intervals (until the required dioptre stabilises). It is therefore important that ready-made near-vision spectacles are affordable enough so they can be replaced when needed.

In my experience, once someone has found a frame design they like, they tend to want to buy a replacement of the same colour and shape. To ensure consistency across orders, I recommend ordering from a verified manufacturer. If you notice a change in the colour or shape between orders, that means the supplier is most likely a reseller, not a manufacturer.

### When ordering

1. Ask the supplier whether the lens has a protective coating, and what materials are used to make the frames. Some materials used to make frames are flexible, while others are more rigid; this affects both the cost and durability of the frame.
2. Confirm the minimum order quantity.
3. Prices should be transparent. Request a price breakdown that separates spectacle costs from import costs.
4. Request a sample batch and test it (see below), but expect minor variation in bulk orders.
5. Before you order a larger quantity, let the manufacturer know that you will be testing the spectacles when they arrive. I recommend you test at least 2% of the main order.

## Basic quality tests that require no specialised equipment

### Frames

1. **Check that the frame material** is what you ordered and paid for by performing a ‘flex’ test:
  - Remove the lenses. Hold the front of the frame and bend it gently.
  - TR90: bends and returns to its shape (Figure 1).
  - Polycarbonate: bends slightly but may deform, snap, or turn white at the bending points (Figure 2).
  - Acrylic: breaks easily or snaps at the bending points.
    **Figure 1:**
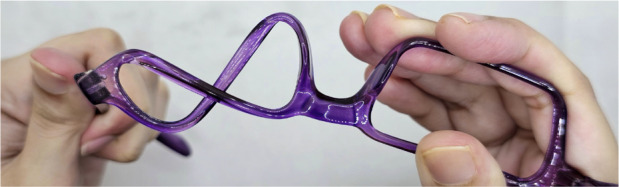
TR90 twists without breaking.

SAMIT SAKIB GORE CC BY-NC-SA 4.0
    **Figure 2:**
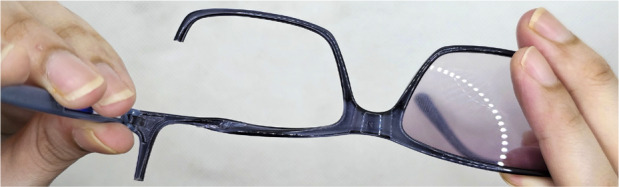
Polycarbonate may snap when twisted.

SAMIT SAKIB GORE CC BY-NC-SA 4.0
2. **Check the screws.** Are they too long, or too short? If so, the spectacles should be rejected. If a screw is too long, it can scratch the skin. If it is too short, the temple will loosen or fall off. Loose or rusty screws are one of the most common reasons spectacles fail in the first few months. If a screw is loose, it can be tightened, but if a screw is rusty, the spectacles should be rejected.
3. **Check the hinges.** If the temples feel wobbly, one or both hinges is loose or unstable. If the hinge has a spring, remove the lenses and gently stretch the arms outward. A good spring hinge should open smoothly and return slightly without resistance. Any cracking noises indicate poor-quality springs. Spring hinges give flexibility, but poor spring quality leads to early breakage.

### Lenses

1. **Check for defects in the lens.** Hold the lens under bright light and rotate slowly. Defects become more visible at different angles. Check for any scratches, bubbles, uneven coating, haze or distortion.
2. **Check the lens fit.** Check if the lens fits in the rim, both with and without movement from the frame (such as opening and closing of the frames.) If the lens moves, it is not mechanically stable.
